# Behavioral and EEGraphic Characterization of the Anticonvulsant Effects of the Predator Odor (TMT) in the Amygdala Rapid Kindling, a Model of Temporal Lobe Epilepsy

**DOI:** 10.3389/fneur.2020.586724

**Published:** 2020-11-05

**Authors:** Polianna Delfino-Pereira, Poliana Bertti-Dutra, Flávio Del Vecchio, José A. Cortes de Oliveira, Daniel de Castro Medeiros, Daniel M. Cestari, Victor R. Santos, Marcio F. D. Moraes, João L. G. Rosa, Eduardo M. A. M. Mendes, Norberto Garcia-Cairasco

**Affiliations:** ^1^Department of Neuroscience and Behavioral Sciences, Ribeirão Preto School of Medicine, University of São Paulo, Ribeirão Preto, Brazil; ^2^Department of Physiology, School of Medicine of Ribeirão Preto, University of São Paulo, Ribeirão Preto, Brazil; ^3^Department of Physiology and Biophysics, Institute of Biological Science Physiology and Biophysics, Federal University of Minas Gerais, Belo Horizonte, Brazil; ^4^Electrical Engineering Graduate Program, Federal University of Minas Gerais, Belo Horizonte, Brazil; ^5^Department of Computer Science, Institute of Mathematics and Computer Sciences, University of São Paulo, São Carlos, Brazil; ^6^Department of Morphology, Institute of Biological Science Physiology and Biophysics, Federal University of Minas Gerais, Belo Horizonte, Brazil

**Keywords:** epilepsy, temporal lobe epilepsy (TLE), amygdala rapid kindling (ARK), seizures, olfaction, 2,5-dihydro-2,4,5-trimethylthiazoline (TMT)

## Abstract

**Background:** Clinical and experimental evidence indicates that olfactory stimulation modulates limbic seizures, either blocking or inducing ictal activity.

**Objective:** We aim to evaluate the behavioral and electroencephalographic (EEGraphic) effects of dihydro-2,4,5-trimethylthiazoline (TMT) olfactory exposure on limbic seizures induced by amygdala rapid kindling (ARK).

**Materials and Methods:** Wistar male rats (280–300 g) underwent stereotaxic surgery for electrode implantation in piriform cortex (PC), hippocampal formation (HIP), and amygdaloid complex (AMYG). Part of the animals was exposed to a saturated chamber with water or TMT, while others had ARK and olfactory exposure prior to the 21st stimulus. Behavioral responses were measured by traditional seizure severity scales (Racine and Pinel and Rovner) and/or by sequential analysis/neuroethology. The electrographic activity of epileptogenic limbic networks was quantified by the occurrence of the first and second EEG afterdischarges, comparing the 1st and 21st stimulus. The spectral analysis [Fast Fourier Transform (FFT)] of the first afterdischarge was performed at the 21st stimulus.

**Results:** TMT olfactory exposure reduced the seizure severity in kindled rats, altering the displayed behavioral sequence. Moreover, TMT decreased the occurrence of first and second afterdischarges, at the 21st stimulus, and altered the spectral features.

**Conclusions:** Both behavioral and EEGraphic evaluations indicated that TMT, a potent molecule with strong biological relevance, in fact, “predator odor,” suppressed the epileptiform activity in limbic networks.

## Introduction

Epilepsy is a hyperexcitable brain disease, characterized by the persistent predisposition to generate epileptic seizures [for full definition, see (1)] and by the neurobiological, psychological, cognitive, and social consequences (2). Seizures are symptomatic manifestations, not necessarily behavioral, of abnormal electrical discharges, derived from either part or the whole central nervous system (3, 4). It is estimated that ~4–10 in every 1,000 people worldwide have epilepsy (3), with temporal lobe epilepsy (TLE) being the most common type in adults (5–10). While significant advances occurred on pharmacological treatments in the last decades, about 20–40% of these patients do not respond to currently available drug treatments (11–14).

A promising alternative in the pool of therapeutic approaches is olfactory stimulation (odor therapy) capable to modulate, block, or even induce seizures. Efron (15) elegantly described the report of a patient who presented seizures with olfactory auras and that the exposure to strong and unpleasant odors, such as pure jasmine, hydrogen sulfide, and others, was able to inhibit the expression of such seizures. Later, the same patient interestingly created a conditioning, where the simple evocation of the olfactory memory was enough to prevent the manifestation of seizures (16). Similarly, experimental data also evidenced promising results, as shown by Ebert and Löscher (17), utilizing the olfactory stimulation with toluene (TOL), an aversive odor, capable of decreasing the seizures' susceptibility in amygdala kindling, even with electrical stimulation 20% above of threshold. The latter report was strongly supported by our experimental findings, confirming the TOL anticonvulsant effects, in the acute and chronic (kindled) audiogenic seizures, in *Wistar Audiogenic Rat* (*WAR*) animals (18). Conversely, the induction of seizures by olfactory stimulation has also been reported, in both animal models and in the clinic (19–22). In fact, the neurophysiological mechanisms of both anti- and pro-convulsant modulations are little understood.

Amygdala kindling (usually electrical) is a clinically relevant animal model of TLE (5, 23–32), widely used in antiepileptic drug screening, including the one sponsored by the NIH/NINDS (27, 29, 30, 33). A fast and effective alternative is the amygdala rapid kindling (ARK) model, which consists of 10 daily electrical stimuli, during 2 consecutive days (34). On the 3rd day, an additional stimulus can be applied to anticonvulsant drug tests (in fully kindled rats) or to study plasticity or memory mechanisms.

It is described that the olfactory stimulation with 2,5-dihydro-2,4,5-trimethylthiazoline (TMT), indeed “*predator odor*,”, induces fear/aversion-like behaviors and triggered rhythmic fast activity (>1 mV; peak frequency, ~16 Hz; mean frequency, ~20 Hz) in the olfactory bulb and the piriform cortex (PC) of rats (35). Recently, it was demonstrated that olfactory inputs modulate freezing behavior in prelimbic prefrontal cortex, an area important for expression of conditioned fear behaviors (36). In fact, the activation of limbic structures, such as PC, amygdaloid complex (AMYG), and hippocampal formation (HIP), is a common feature when studying both “fear/aversion-related” networks [for revision about brain circuits involved in aversion-related processing, see (37)] and “seizure related” networks. Thus, considering the evidence of the control or blockade of seizures by olfactory stimulation in rodents and patients, our main objective was to evaluate the behavioral and electroencephalographic (EEGraphic) effects of TMT olfactory exposure on limbic seizures induced by ARK.

## Materials and Methods

### Ethics Statement

All experimental protocols were designed according to recommendations for animal experimentation of the Brazilian Society for Neuroscience and Behavior and from the Commission for Ethics in Animal Experimentation (CETEA), at the University of São Paulo (protocol number: 200/2011).

### Animals

Male Wistar rats (280–300 g) were kept in individual Plexiglas cages, at the Vivarium of the Ribeirão Preto School of Medicine, placed at the Department of Physiology, under controlled environmental conditions of light (lights on at 06:00 h; lights off at 18:00 h), temperature (23 ± 2°C), and food and water *ad libitum*. After the experimental procedures (described below), the rats were euthanized.

### Stereotaxic Surgery

All animals were anesthetized with Thiopentax—thiopental sodium 4% [30 mg/kg, intraperitoneal injection (ip)] with doses of maintenance (0.1 ml) with combination of anesthetics Ketamine (0.06 mg/kg, ip; Agener Union Animal Health—Embu Guaçu, SP, Brazil) and Xylazine (0.04 mg/kg; ip; Bayer Animal Health—São Paulo, SP, Brazil). In addition, in the scalp, subcutaneous anesthetic composed of 2% lidocaine hydrochloride containing epinephrine (Astra—Naucalpan, Mexico; 5 mg/kg) was injected before incision.

Bipolar electrodes made of twisted stainless steel (0.005 in.) and Teflon-coated (0.007 in.) wires (Model 791400, AM Systems Inc., Carlsborg, WA, USA) were stereotactically implanted in the left PC (AP: Bregma, ML: −5.0 mm, DV: −8.2 mm) and HIP (AP: −6.3 mm, ML: −4.5 mm, DV: −4.5 mm). For stimulation and record, we used a tripolar electrode in the basolateral amygdala (AP: −2.3 mm, ML: −4.7 mm, DV: −7.1 mm) Additionally, a ground wire and four stainless steel screws (Ø 1.5 mm; Fine Science Tools, Heidelberg, Germany) were implanted. The electrodes, ground wire, and screws were fixed to the bone with zinc cement (S.S. White—Rio de Janeiro, RJ, Brazil) and subsequently welded (two first items) to a connector (RJ11cat6), which in turn was fixed to the rat skull with acrylic resin. After surgery, the animals received intramuscular veterinary pentabiotic (0.05 ml; Fort Dodge Animal Health LTDA—São Paulo, SP, Brazil).

The animals remained in recovery for a period of 3–7 days, with daily 10-min habituation sessions in the last 3 days, in order to minimize responses to novelty.

### Experimental Procedure

The ARK followed the model proposed by Foresti et al. (34), consisting of the application of 20 electrical stimuli, ten times a day (morning period), for 2 consecutive days, with interstimulus interval of 30 min. On the 3rd day, 24 h after the 20th stimulus, the animals were submitted to the olfactory stimulus and immediately after received the last electrical stimulus (the 21st stimulus or test stimulus). During the sessions, the behavior and electroencephalogram (EEG) were recorded for posterior analysis.

Four groups of animals were studied: (1) No Stimulus and Water: sham rats exposed only water, (2) Stimulus and Water: rats submitted to ARK and to water 20 s before the test stimulus, (3) No Stimulus and TMT: sham rats exposed only to TMT, and (4) Stimulus and TMT: rats submitted to ARK and to TMT 20 s before the test stimulus.

The No Stimulus groups (sham kindling) consisted of rats exposed only to odorants, which were manipulated equally as the rats that received the ARK protocol, but with no electrical stimulation.

### Electrical Stimulation

The EEG afterdischarge threshold was determined through stimulation delivered to the AMYG (GRASS-S88 stimulator; West Warwick, USA), with an initial current of 100 μA, during 2 s and progressive increases of 100 μA, every 5 min, until the presence of the first afterdischarge, according to Foresti et al. (34) and Ebert and Löscher (17). Independent of the established threshold, the parameters used for stimulation were trains of biphasic square waves, with a constant intensity of 500 μA, 1-ms pulses, delivered at 60 Hz, for a period of 10 s (23, 34).

Electrophysiological signals were recorded 5 min before and after each stimulus, with the exception of the 21st stimulus (with a duration of 20 s, for more details, see section Olfactory Stimulation), using a CyberAmp (Axon Instruments), which transduced and conditioned the signals. The latter were digitized (MP100, Biopac Systems) and recorded with the Acqknowledge software (Biopac Systems; Santa Barbara, USA). The parameters for EEG recording were total gain of 2,000 times, low-pass filter at 1,000 Hz, high-pass filter at 0.1 Hz, sampling rate at 2,000 Hz, and notch filter in 60 Hz, similar to Foresti et al. (34).

### Olfactory Stimulation

The olfactory stimulus occurred in the same chamber of the electrical stimulus, for this, 5 μl of liquid (water or TMT) was soaked under a piece of filter paper and kept inside the chamber completely closed during 30 min [temporal and chemical parameters assessed in a previous study (18)]. Subsequently, the filter paper was removed and the animals were placed inside the chamber. After 20 s, the 21st electrical stimulation was applied and the behavioral and EEGraphic parameters were recorded. Then, animals were removed and the exhaustion system was turned on for 30 min.

### Behavior Analysis

Limbic index (LI) for seizures was classified according to Racine's scale (38) modified by Pinel and Rovner (39) (Table 1). The animals were considered fully kindled when presented at least two class 4 seizures or one class 5 seizure during the entire protocol.

**Table 1:** Severity index with behavioral descriptions according to Limbic index (LI) or Racine scale (38) modified by Pinel and Rovner (39).

0 = Immobility
1 = Facial automatism
2 = Head myoclonia
3 = Forelimbs myoclonia
4 = Rearing
5 = Rearing and falling
6 = More than three fallings
7 = Running and/or jumping (Wild running)
8 = Tonic-clonic seizures

**Figure 1:**
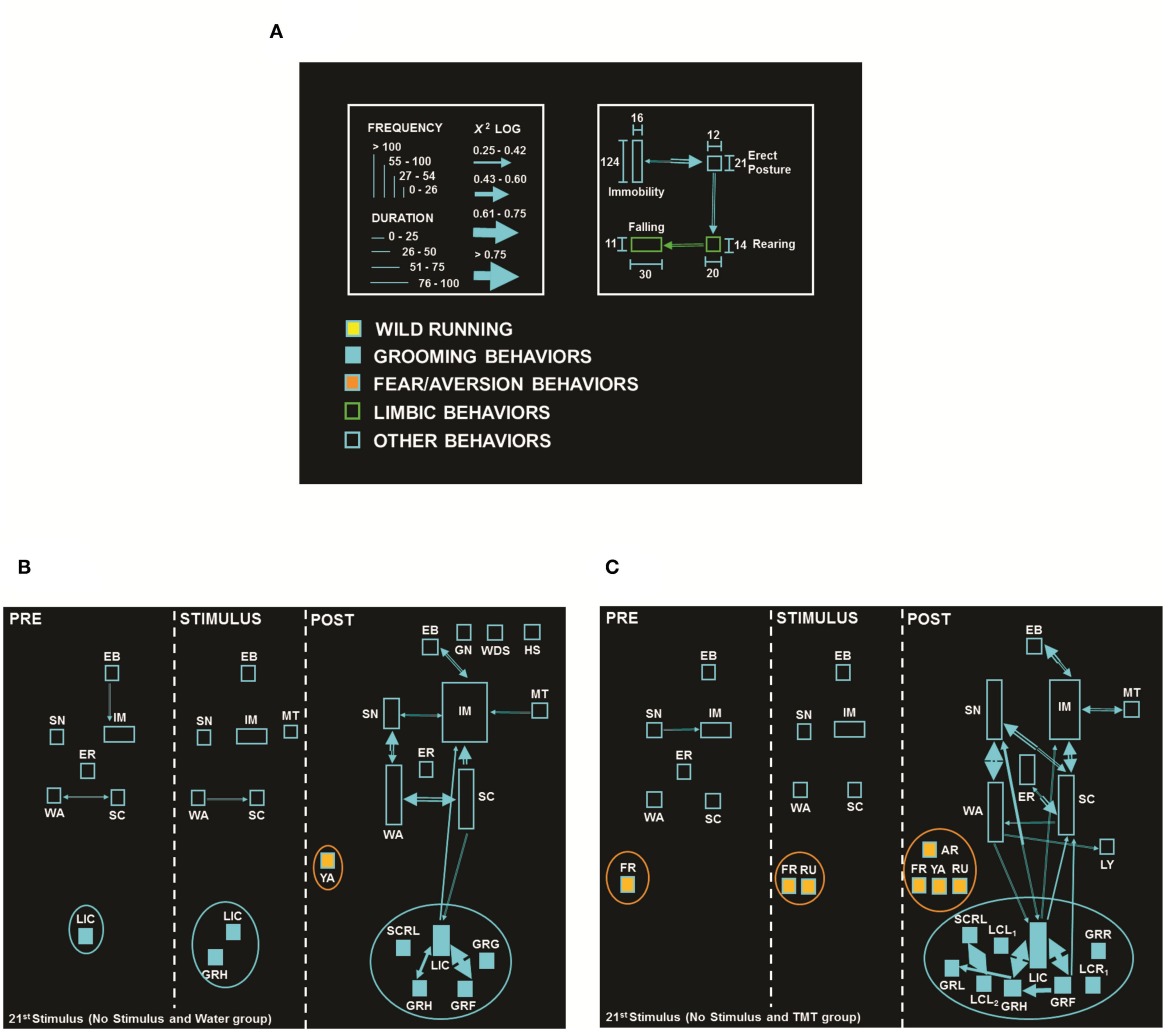
**(A)** Flowchart calibration pattern: each behavior is represented as a rectangle, its height corresponds to behavioral frequency, and the base corresponds to behavioral duration. Statistical interactions between pairs (dyads) are proportional to the width of the arrows that link them. The colors used in the rectangles qualitatively identify behavioral categories or classes (clusters). The color of the arrows is determined by the starting point of each dyad (40). **(B)** Flowchart of behavioral response from No Stimulus and Water group (*N* = 8), with the presence of fear/aversion behaviors: Yawning (YA, orange ellipse). **(C)** Flowchart of behavioral response from No Stimulus and TMT (*N* = 8), with the presence of fear/aversion behaviors: Arousal (AR), Freezing (FR), Running (RU), and YA (orange ellipse). In each flowchart, the PRE period (first cell) corresponds to the olfactory stimulation period with a duration of 20 s, the STIMULUS period (middle cell) corresponds to the simulation period of the electrical stimulus with a duration of 10 s, and the POST-period (third cell) corresponds to the period after the simulation of the electrical stimulation, with a duration of 5 min. Behavioral dictionary and acronyms: EB, Eye Blinking; ER, Erect Posture; GN, Gnawing; GRF, Grooming of Face; GRG, Grooming of Genitals; GRH, Grooming of Head; GRL, Grooming of Body, Left; GRR, Grooming of Body, Right; IM, Immobility; LCL_1_, Licking of Claws Left, Forelegs; LCL_2_, Licking of Claws Left, Hindlegs; LCR_1_, Licking of Claws Right, Forelegs; LIC, Licking of Claws; LY, Lying Posture; MT, Masticatory Movements; SC, Scanning; SCRL, Scratching of Body, Left; SH, Head Shaking; SN, Sniffing; WA, Walking; WDS, Wet Dog Shaking (the complete list of acronyms is summarized in Supplementary Table 1).

In addition, the neuroethological analysis was made by observation and recording of the behavioral sequences presented second by second, during the observation period, based on a Behavioral Glossary (Supplementary Table 1). After recording, the data collected were analyzed using the ETHOMATIC statistical software (40), which provided the frequency of occurrence, the mean duration of each behavior, and the statistical interaction between behavioral pairs (dyads), and the resulting flowcharts were graphically represented, using the calibration standard illustrated in Figure 1A (see more details in the legend). Three periods were evaluated: (1) PRE—basal period or 5 min before the electrical stimulus (except for the 21st stimulus, with a duration of 20 s); (2) STIMULUS—during the electrical stimulus with a duration of 10 s, and (3) POST−5 min after electrical stimulation (or post-ictal period).

### EEGraphic Analysis

The electrographic seizure signal was set as high amplitude (at least two times) over the background, present at least 3 s or more (34, 41). The ictal activity was initially measured by the first and second EEG afterdischarge occurrence, during the 1st and 21st stimulus. The presence or absence of afterdischarges was quantified for each electrically stimulated animal (Stimulus and Water and Stimulus and TMT). The presence–absence distribution was further analyzed (χ^2^ test) separately for each group. If the odor influences the seizure occurrence, a statistical difference is expected in the presence–absence incidence pattern toward a decrease of EEG afterdischarges episodes, i.e., low presence and larger absence numbers. In addition, EEG spectral analysis was performed utilizing Fast Fourier Transform (FFT), as follows.

#### Fast Fourier Transform

FFT spectral analysis was calculated by the median frequency, peak power, the frequency at the peak power, and total power, for all groups. For descriptive purposes, the median frequency was defined as the frequency value that up to that frequency sums 50% of the energy signal. Total power is the amount of energy/power contained in the signal. Peak power and frequency at the peak power are the maximum value of the power for a given frequency and its frequency.

For each channel (PC, AMYG and HIP), the first afterdischarge (for Stimulus groups) was extracted after the 21st electrical stimulation, while for the No Stimulus groups, we considered for analysis the whole signal after ~30 s (to establish the basal period). For each signal, the FFT was computed extracting the four features previously described, considering the following frequency bands: delta (≤4 Hz), theta (4–8 Hz), alpha (8–14 Hz), beta (14–30 Hz), gamma (30–100 Hz), as well as the whole spectrum. In the whole spectrum, we considered frequencies below 100 Hz to avoid high-frequency contamination artifact. In the case of the Stimulus groups, we performed a filtering process to remove the influence of capacitor discharges. The filtering process chopped off the beginning part of the first afterdischarge with a mean value below the basal level. The signals were analyzed using Python (3.7.2, programming language) software and the NumPy (1.15.4), SciPy (1.2.0), and Matplotlib (3.0.2) libraries.

### Statistical Analysis

LI was analyzed with one-way ANOVA, in order to evaluate the effectiveness of ARK, and Mann–Whitney test to verify the TMT effect on seizures.

Data from the neuroethological analysis, as cited, indicated the frequency of occurrence of each item and its duration. Moreover, it provided analysis of statistical interaction between behavioral pairs (dyads) by the analysis of chi-square (χ^2^), with interactions being considered statistically significant when χ^2^ ≥ 3.84, log χ^2^ ≥ 0.25; *p* < 0.05 (40).

The occurrence (presence or absence) of first and second EEG afterdischarges was also statistically analyzed by χ^2^ test, comparing the 1st and 21st stimulus for animals of the groups: Stimulus and Water, and Stimulus and TMT. The statistical evaluation for the FFT analysis was performed by Mann–Whitney test.

All values were expressed as means ± standard error of the mean (SEM). Data were analyzed using GraphPad Prism (7.0) and Matlab (2016) software. Significance level was set at *p* ≤ 0.05.

## Results

**Figure 2:**
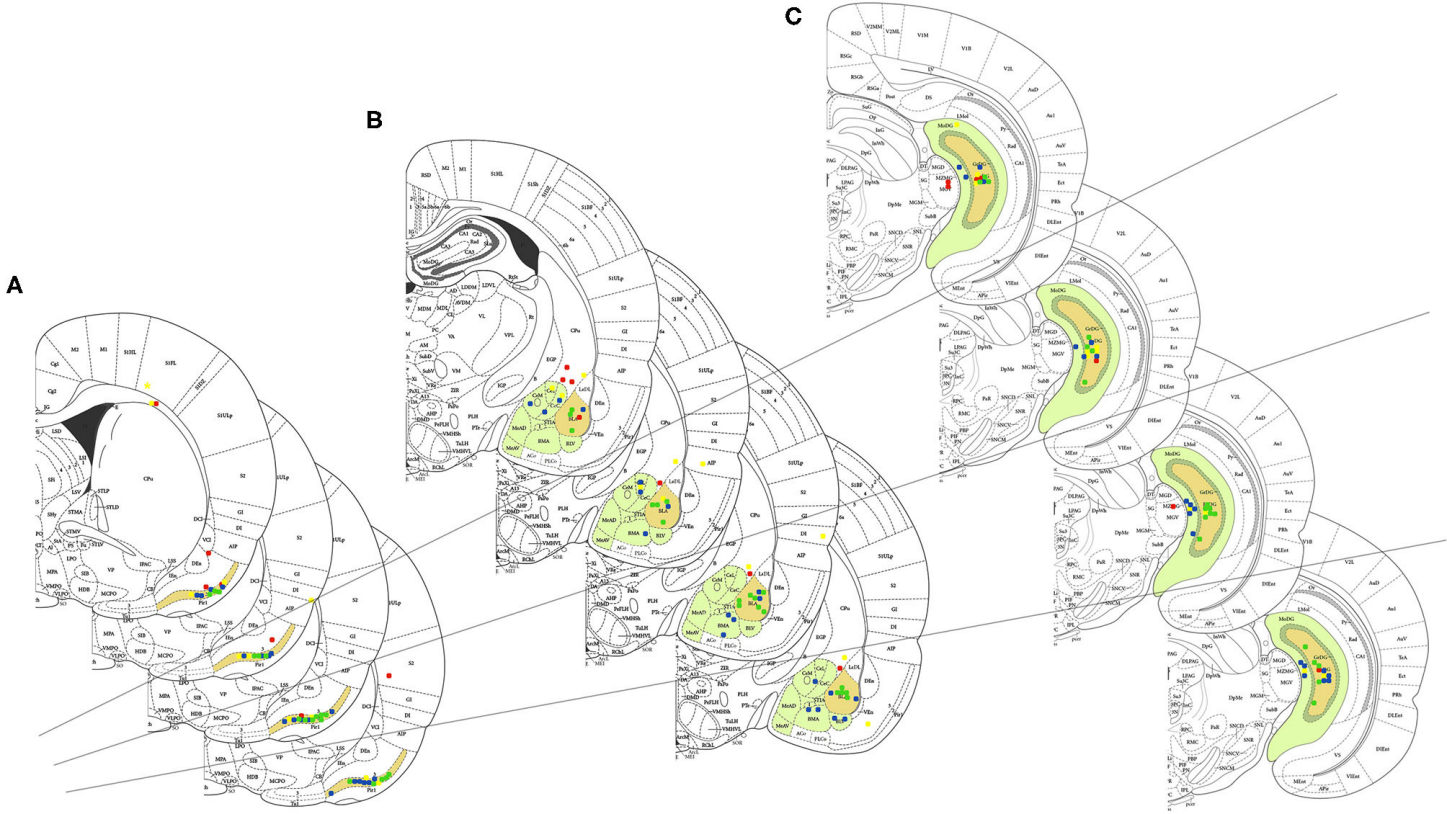
General electrode positioning data in the groups: No Stimulus and Water (1st plate or superior plate), Stimulus and Water (2nd plate), No Stimulus and TMT (3rd plate), and Stimulus and TMT (4th plate or inferior plate), with schematic design in the following areas: **(A)** piriform cortex, **(B)** amygdaloid complex, and **(C)** hippocampal formation, all in the left hemisphere of the 57 animals that were implanted with electrodes in the stereotaxic surgery. The following are represented in closed circles with different colors: red, the animals that hit only one of the cerebral areas described above; yellow, the animals with accuracy in two brain areas (piriform cortex, amygdaloid complex, and/or hippocampal formation); blue, the animals with electrode positioned in the piriform cortex, amygdaloid complex, and hippocampal formation; and green, the animals with the positioning in the piriform cortex, basolateral amygdala, and hilus of the gyrus dentate.

Only animals with the correct position of electrodes and kindled were included in the study. Figure 2 shows the positioning of the electrodes in the following areas: PC (1), AMYG (2), and HIP (3), for the following groups: No Stimulus and Water (*N* = 8), Stimulus and Water (*N* = 8), No Stimulus and TMT (*N* = 8), and Stimulus and TMT (*N* = 10), of the 57 animals implanted during stereotaxic surgery.

### TMT Decreased the Severity of Limbic Seizures

**Figure 3:**
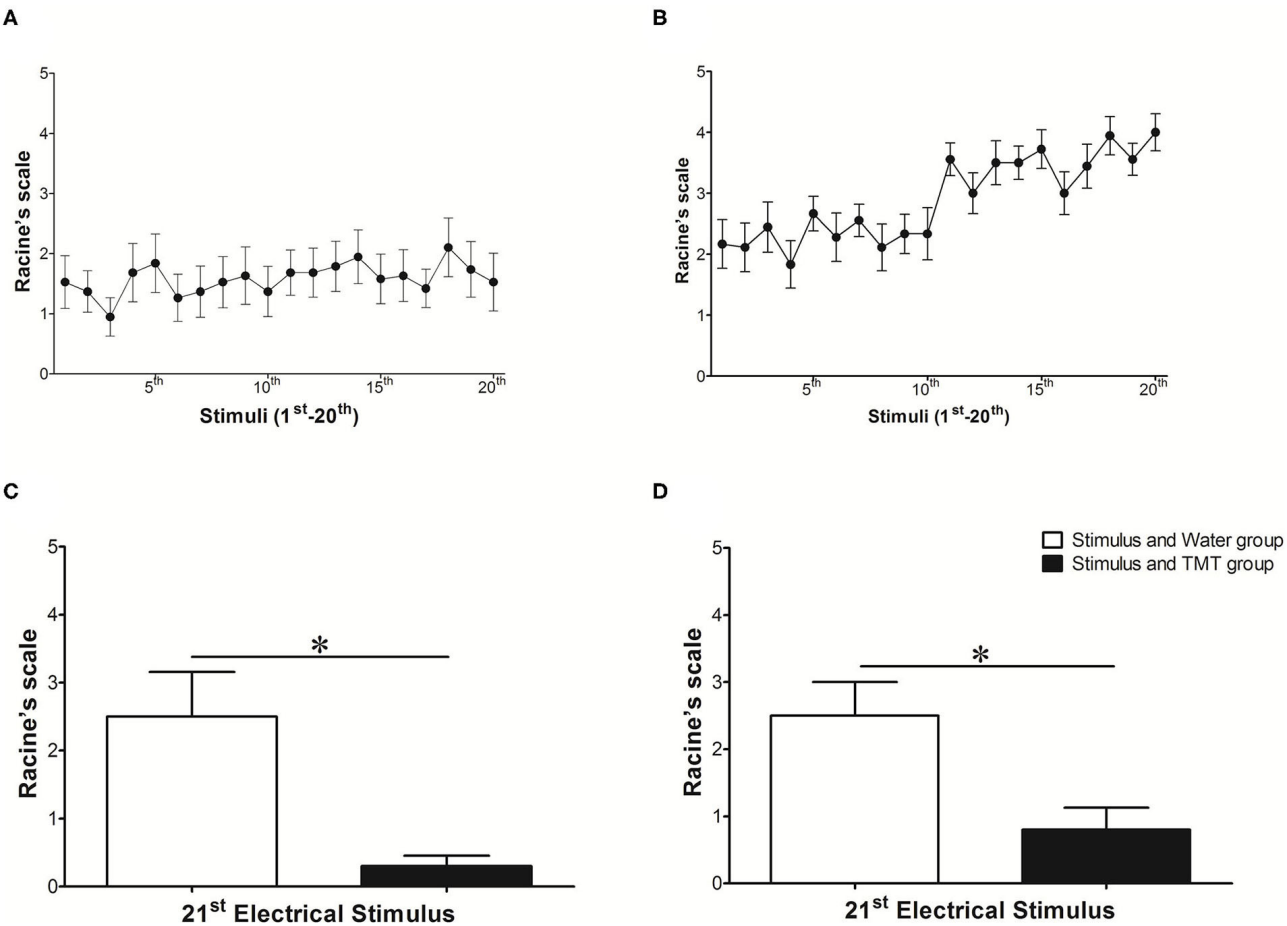
Behavioral classification of seizures observed during the evolution of amygdala rapid kindling: **(A)** STIMULUS (1st−20th stimulus) corresponds to period of electrical stimulus with a duration of 10 s, for animals of both stimulated groups (*N* = 18). **(B)** POST (1st−20th stimulus) corresponds to period after electrical stimulation (or post-ictal period) with a duration of 5 min, also with animals of both stimulated groups (*N* = 18). In **(C)** and **(D)**, the comparison of the means obtained by Racine's scale (38) at the 21st stimulus, with the previous intervention of the water in the Stimulus and Water group (*N* = 8, white bar), vs. TMT in the Stimulus and TMT group (*N* = 10, black bar) obtained during the **(C)** STIMULUS period (10 s) and in the **(D)** POST-stimulus period (5 min). In the *Y* axis, the mean severity index for limbic seizures according to Racine (38) reached in each stimulus indicated by the *X* axis **(A,B)** or in each group **(C,D)**. **(A,B)** One-way ANOVA, Friedman test, and Dunn's post-test (with difference between stimuli indicated in Supplementary Table 2). **(C,D)** Mann–Whitney test, *p < 0.05.

The evolution of ARK (1st−20th stimulus) is illustrated in Figures 3A,B, for animals of both stimulated groups (*N* = 18, Stimulus and Water, and Stimulus and TMT) during the stimulus period (A) and post-stimulus period (B). It is possible to verify the expected evolution of ARK with a natural and progressive development of kindling, with statistically significant differences along the stimuli (Supplementary Table 2). However, the exposure of kindled rats to TMT significantly reduced the severity of seizures at the 21st stimulus [Racine's scale or LI (Figures 3C,D)]. In addition, TMT (Stimulus and TMT) significantly reduced the LI compared to the 20th stimulus (mean of 4.00 to 0.5 in the TMT presence; *p* < 0.0001; Mann–Whitney test), but there was no difference for the Stimulus and Water group (mean of 3.50 to 2.50 in the water presence; *p* > 0.05; Mann–Whitney test) in the POST-period. More specifically, at the 21st stimulus, in the Stimulus and TMT group, 5 out of 10 rats (50%) did not present seizures (LI = 0). In the other five rats (50%) in which the seizures were observed, three (30%) presented LI equal to class 1 (30%) and only two (20%) showed seizures varying from classes 2 or 3.

Thus, the ARK protocol induced behavioral seizure progression, resulting in complex partial seizures with secondary generalization. The exposure of Wistar rats after ARK to TMT reduced significantly limbic seizures (Racine's scale or LI). This criterion, besides being validated, is frequently used in the evaluation of therapeutic interventions (27, 32, 42–44).

### Behavioral Sequences Confirmed the Suppressive Effect of TMT in Limbic Seizures

The neuroethological analysis represented in Figures 1B,C illustrates the flowcharts of the No Stimulus and Water (B, *N* = 8) vs. No Stimulus and TMT (C, *N* = 8) groups, both during the 21st stimulus. Figure 1C shows the flowchart of the No Stimulus and TMT group evidencing the behaviors (rectangles) and their statistical interactions (arrows) after exposure to TMT (Figure 1C), with the expression of fear/aversion-like reactions, such as Freezing (FR), found in the 1st cell. Also, during the STIMULUS period (in this case, without stimulus, 2nd cell), the animals remained with the same fear/aversion behavior, in addition to Running (RU). However, in the POST-period, TMT triggered more complex fear/aversion-like reactions, such as FR, RU, Arousal (AR), and Yawning (YA), which activated natural exploration for clues that indicated the location of the predator, demonstrated by higher number of behaviors and interactions, added to higher frequency of Sniffing (SN) and Erect Posture (ER), in comparison to its control (No Stimulus and Water group, Figure 1B).

**Figure 4:**
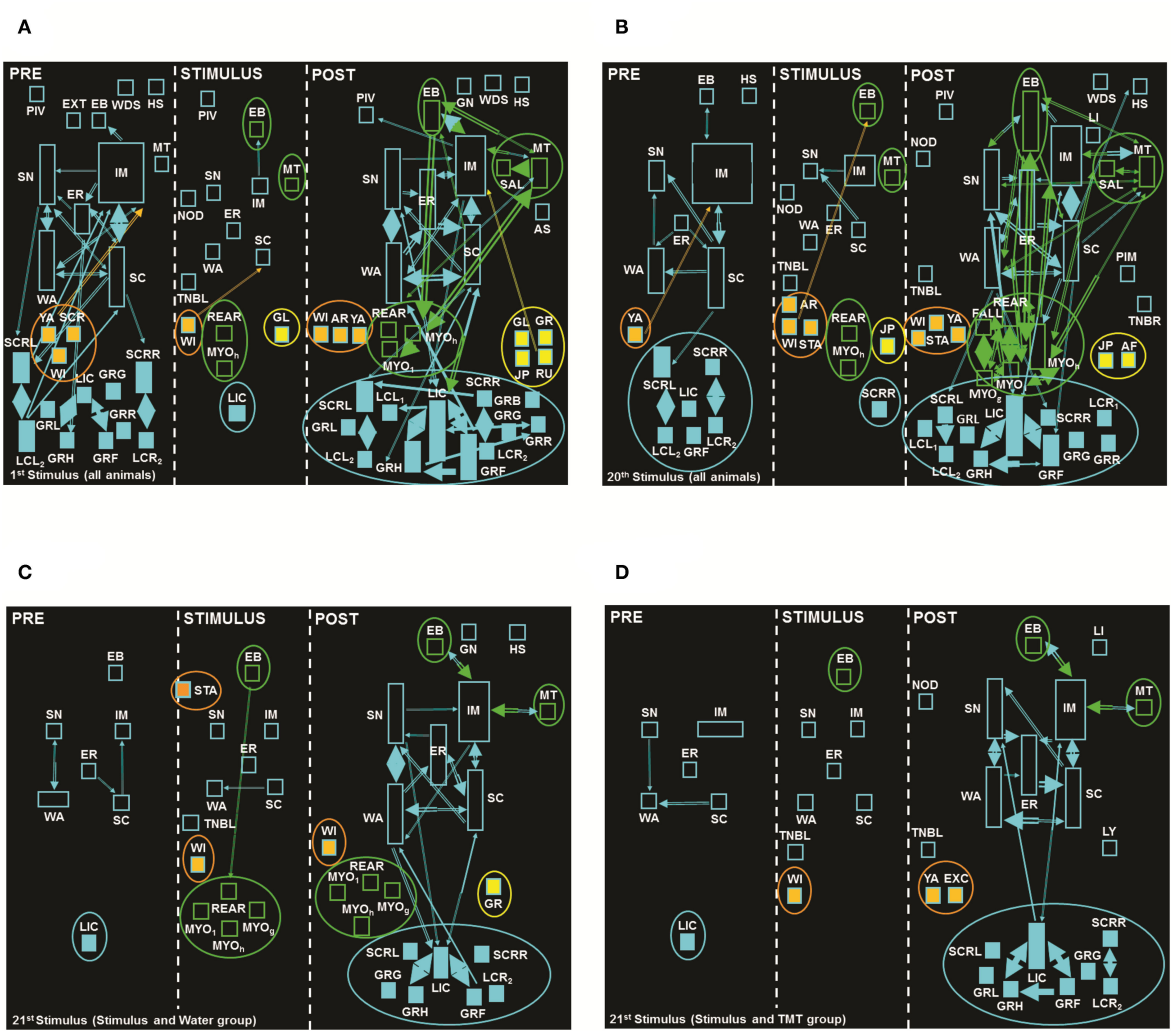
Flowcharts with the sum of seizures from the **(A)** 1st and **(B)** 20th stimuli of animals both stimulated groups. **(C)** and **(D)** are the flowcharts from the 21st stimulus: **(C)** Stimulus and Water, and **(D)** Stimulus and TMT. In the first cell (left) of each stimulus, the PRE period with 5 min before stimulus (exception: 21st stimulus with a duration of 20 s); in the second cell (middle), the STIMULUS period with 10 s, and finally in the third cell (right), the POST-period also with a duration of 5 min after the stimulus. Calibration details such as rectangles, arrows, and colors; see Figure 1A. Behavioral dictionary and acronyms: AF, Atonic Falling; AR, Arousal; AS, Abdominal Spasms; EB, Eye Blinking; ER, Erect Posture; EXT, Extension; FALL, Falling; GL, Gyrating, Left; GN, Gnawing; GR, Gyrating, Right; GRB, Grooming; GRF, Grooming of Face; GRG, Grooming of Genitals; GRH, Grooming of Head; GRL, Grooming of Body, Left; GRR, Grooming of Body, Right; IM, Immobility; JP, Jumping; LCL_1_, Licking of Claws Left, Forelegs; LCL_2_, Licking of Claws Left, Hindlegs; LCR_1_, Licking of Claws Right, Forelegs; LI, Licking; LIC, Licking of Claws; MT, Masticatory Movements; MYO_1_, Myoclonus Spasms, Forelegs; MIO_g_, Generalized Myoclonus; MYO_h_, Myoclonus Spasms, Head; NOD, Nodding; PIM, Postictal Immobility; PIV, Pivoting; REAR, Rearing; RU, Running; SAL, Salivation; SC, Scanning; SCRL, Scratching of Body, Left; SCRR, Scratching of Body, Right; SH, Head Shaking; SN, Sniffing; STA, Startle; TNBL, Tonic Neck and Body Turning Left; TNBR, Tonic Neck and Body Turning Right; WA, Walking; WDS, Wet Dog Shaking; WI, Withdraw; YA, Yawning (the complete list of acronyms is summarized in Supplementary Table 1).

Figure 4 shows the behavioral effect observed in the 1st (A) and 20th (B) electrical stimuli, for both stimulated groups (Stimulus and Water, and Stimulus and TMT, *N* = 18). In the PRE period of 1st stimulus (Figure 4A, 1st cell), it was possible to verify a clear heterogeneity, especially for exploratory and grooming (self-cleaning) behavioral items. In this context, animals expressed considerably exploratory behaviors that include ER, Immobility (IM), Scanning (SC, head movements), SN, and Walking (WA). There was also a number of variations (qualities) of grooming (self-cleaning) behaviors such as Grooming of Face (GRF), Grooming of Genitals (GRG), Grooming of Head (GRH), Grooming of Body on the Left (GRL), Grooming of Body on the Right (GRR), Licking of Claws (LIC), Licking of Claws Forelegs Left (LCL_1_), Licking of Claws Forelegs Right (LCR_1_), Scratching of the Body Left (SCRL), and Scratching of the Body Right (SCRR), with strong interactions between some of these behaviors, which characterizes a repetitive pattern of the sequence of these items, in other words, grooming (self-cleaning) clusters.

During the STIMULUS period (Figure 4A, 2nd cell) we observed the presence of some isolated behaviors, with only two interactions between the pairs [IM—Eye Blinking (EB) and Withdrawal (WI)—SC]. In addition, there was the presence of seizure behaviors such as EB, Masticatory Movements (MT), and Rearing (REAR), with LI equal to four, added to the behavior Gyri on the Left (GL, asymmetric posture and turning).

In the last cell, POST-period (Figure 4A, 3rd cell), there was an increase in the number of behaviors and dyadic (pair of behaviors) interactions, with the predominance of behaviors named as Other including exploration and grooming (self-cleaning) behaviors. The presence of clusters formed by quite localized limbic behaviors, in fact orofacial automatisms, such as EB, MT, Salivation (SAL), Myoclonic Spasms of Forelegs (MYO_1_), Myoclonic Spasms of the Head (MYO_h_), and REAR, was also clear, in addition to typical seizure behaviors such as GL, Gyri on the Right (GR), Jumping (JP), and RU, besides some fear/aversion-related behaviors, such as AR, WI, and YA.

In the PRE period of the 20th stimulus (Figure 4B, 1st cell), there was a reduction in the number of behavioral items presented by the animals, with the presence of exploratory and grooming (self-cleaning) behaviors, in addition to EB, Head Shaking (HS), and YA. These behaviors in general presented frequency and duration less than in the same period of the 1st Stimulus (Figure 4A, 1st cell), with the exception of IM, with increased importance in proportional time. Moreover, there was a reduction in behavioral interactions during this period.

During stimulation (STIMULUS period, Figure 4B, 2nd cell) in the 20th stimulus, exploratory behaviors (SC, EP, IM, SN, and WA) and other new ones were detected, when compared to the same period of the 1st stimulus (Figure 4A, 2nd cell), such as AR, SCRR, and Startle (STA), in addition to the limbic behaviors of EB, MT, MYO_h_, and REAR, together with the mesencephalic behavior of JU. Still, in this period, there were three behavioral interactions, one more than at the same period of the 1st stimulus (Figure 4A, 2nd cell). In the period after the 20th stimulus (POST, Figure 4B, 3rd cell), there was the presence of the same limbic behaviors, when compared with the 1st stimulus (Figure 4A, 3rd cell), such as EB, MT, MYO_h_, MYO_1_, SAL, and REAR, plus Generalized Myoclonus (MYO_g_) and Falling (FALL). Two additional behaviors were also observed: Atonic Falling (AF) and JU, and still, in this situation, Post-ictal Immobility (PIM) was also present, naturally associated to the post-seizure periods.

Finally, in the PRE period of the 21st stimulus for both stimulated groups, we identified the presence of five exploratory behaviors (ER, IM, SC, SN, and WA) and an isolated grooming (self-cleaning) behavior (LIC; Figures 4C,D, 1st cells). The animals exposed to TMT (Figure 4D, 1st cell) showed an increased proportional time for the IM item, while the animals submitted to water (Figure 4C, 1st cell) still presented EB. There were only three interactions in the Stimulus and Water group (Figure 4C, 1st cell) vs. two behavioral interactions in the Stimulus and TMT group (Figure 4D, 1st cell).

During the 21st stimulus (STIMULUS period), the Stimulus and Water group (Figure 4C, 2nd cell) presented the same behaviors observed for the same period of the 20th stimulus (Figure 4B, 2nd cell), with the exception of AR, JP, MT, and Nodding (NOD), in addition to the presence of limbic behaviors, such as MYO_1_ and MYO_g_. For the Stimulus and TMT group (Figure 4D), during the stimulation (STIMULUS period, 2nd cell), the presence of a single seizure behavior, EB, was observed.

In the 21st stimulus posterior period (POST, 3rd cells, Figures 4C,D, *N* = 8 and *N* = 10, respectively) we observed a reduction of behavioral items in comparison to the same period of the 20th stimulus (Figure 4B, 3rd cell; *N* = 18). These data can be explained by the lower number of animals included in the analysis of the 21st stimulus. For the Stimulus and Water group (Figure 4C, 3rd cell), we observed limbic behaviors such as MT, MYO_h_, MYO_1_, MYO_g_, and REAR (LI = 4), as well as the item GL. For the same period of the Stimulus and TMT group (Figure 4D, 3rd cell), it was possible to observe an expressive reduction in the number of limbic behaviors, with the presence of only two of them: EB and MT (LI = 1). Also, there was the important cluster formation among grooming (self-cleaning behaviors, Figure 4D, 3rd cell) behaviors, in relation to the same period of its control (Figure 4C, 3rd cell).

Comparing the behaviors expressed by the groups: No Stimulus and TMT (Figure 1C) vs. Stimulus and TMT (Figure 4D), the PRE and STIMULUS periods of both groups were similar. They had exploratory behaviors, but the second group (Figure 4D) presented greater duration of the IM item and absence of FR and RU, in contrast to the No Stimulus and TMT group (Figure 1C), where those behaviors were present. In the POST-period, it is possible to observe the presence of typical fear/aversion behaviors (AR, FR, and RU) for the No Stimulus and TMT group (Figure 1C), while for Stimulus and TMT (Figure 4D), there was the behavioral expression of another item of fear/aversion such as Excretion of Feces (EXC).

In summary, we observed predominantly the presence of exploratory and grooming (self-cleaning) behaviors in the PRE (s) periods of all stimuli and groups (Figures 4A–D, 1st cells), even with variations in frequency and duration. With regard to the STIMULUS period (Figures 4A–D, 2nd cells), we observed, in addition to exploratory behaviors, those described as Other, as well as limbic behaviors already present at the 1st stimulus (MT, EB and REAR, Figure 4A, 2nd cell), which along the ARK illustrated the natural increase in severity of the limbic seizures, as shown at the 20th stimulus (EB, MT, MYO_h_, and REAR, Figure 4B, 2nd cell). In the 21st stimulus, the control group (Figure 4C, 2nd cell) presented EB, MYO_h_, MYO_1_, MYO_g_, and REAR, while the experimental group (Figure 4D, 2nd cell) displayed only EB.

In the POST-period, it was possible to notice an increase in limbic behaviors in the 20th stimulus (EB, MT, SAL, MYO_h_, MYO_1_, MYO_g_, REAR, and FALL; Figure 4B, 3rd cell) compared to the 1st stimulus (EB, MT, SAL, MYO_h_, MYO_1_, and REAR; Figure 4A, 3rd cell). In the 21st stimulus, the Stimulus and Water group (Figure 4C, 3rd cell) presented one procursive (asymmetric turning) behavior (GR) and six limbic behaviors (EB, MT, MYO_h_, MYO_1_, MYO_g_, and REAR; LI = 4). On the other hand, the Stimulus and TMT group (Figure 4D, 3rd cell) expressed only two limbic behaviors (EB and MT; LI = 1). Another important aspect observed during the POST-period at the 21st stimulus was the presence of stronger interactions between grooming (self-cleaning) behavioral pairs for the Stimulus and TMT group (Figure 4D, 3rd cell), compared to the control group (Figure 4C, 3rd cell).

The overall behavioral findings indicated that TMT triggered fear/aversion-like reactions and activated natural exploration for clues that indicated the possible location of the predator. Furthermore, the ARK model effectively increased the severity of limbic seizures along the stimuli (1st−20th). In particular, detailed behavioral (neuroethological) analysis confirmed the suppressor effect of TMT in limbic seizures (21st stimulus).

### TMT Decreased the Occurrence of First and Second EEG Afterdischarges

**Figure 5:**
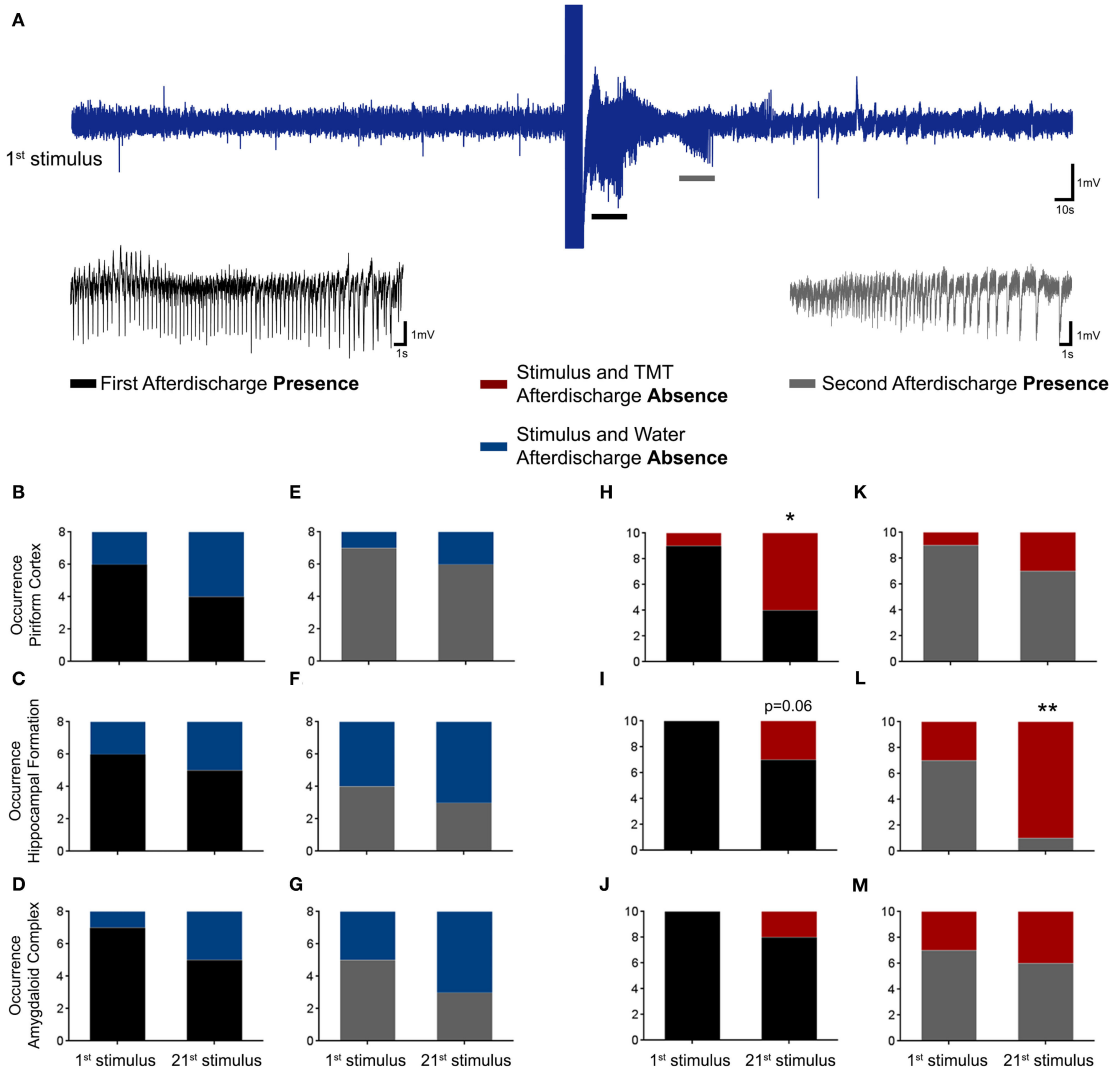
First and second EEG afterdischarges occurrence at the 1st and 21st stimulus for Stimulus and Water, and Stimulus and TMT groups. Upper panel **(A)** demonstrates a representative raw signal from the 1st stimulus. The inset depicts a typical first (black trace) and second (gray trace) polyspike afterdischarges. The lower panel (graphics) shows the quantification of the afterdischarges presence and absence for the neural structures: piriform cortex, hippocampal formation, and amygdaloid complex. First and second afterdischarges analyses for Stimulus and Water group [first afterdischarge **(B–D)**; second afterdischarge: **(E–G)**], and Stimulus and TMT group [first afterdischarge: **(H–J)**; second afterdischarge: **(K–M)**] are presented in sequence. χ^2^ test, *p < 0.05 and **p < 0.01.

The EEG afterdischarge evaluation (Figure 5) demonstrated a significant reduction in electrographic seizure presentation for the TMT animals (Stimulus and TMT group, *N* = 10) but none for the Stimulus and Water group (*N* = 8). Figure 5A shows a typical raw signal of the first stimulation (1st stimulus), highlighting the first (black trace) and second (gray trace) afterdischarge. The lower panel presents the statistical analysis of the afterdischarge occurrence (presence or absence distribution) for the Stimulus and Water group (left panel; 1st and 21st stimuli) and the Stimulus and TMT group (right panel; 1st and 21st stimuli). The animals from the Stimulus and Water group demonstrated no significant distinction (Figures 5B–G). Nevertheless, the Stimulus and TMT group presented a statistical difference in the presence–absence distribution for the 21st stimulus compared with the 1st stimulus, indicated by the reduction of afterdischarge presence numbers and the increase of the absence measures for the neural structures PC [first afterdischarge $\chi_{\left(1\right)}^{2}$ = 5.5, *p* < 0.05—Figure 5H] and HIP [first $\chi_{\left(1\right)}^{2}$ = 3.5, *p* = 0.06 and second afterdischarges $\chi_{\left(1\right)}^{2}$ = 7.5, *p* < 0.01—Figures 5I,L, respectively].

### EEGraphic Activity Altered With TMT

**Figure 6:**
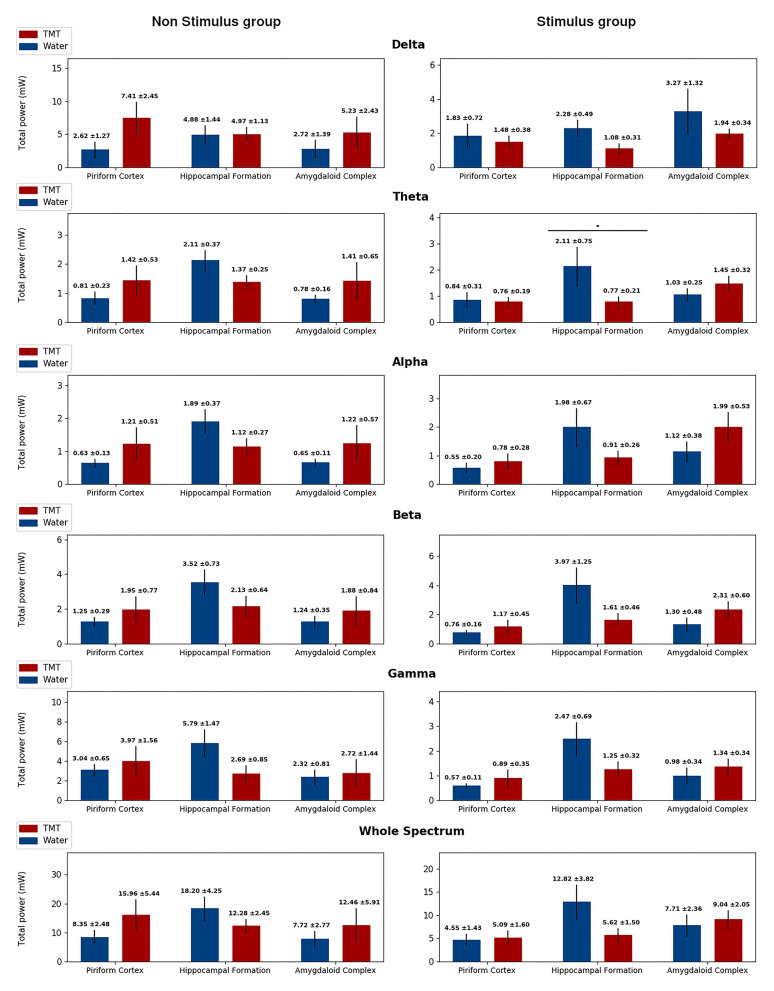
Total power separated in band frequencies for the No Stimulus groups (No stimulus and Water in blue, and No stimulus and TMT in red) on the right column, and from the Stimulus groups (Stimulus and Water in blue, and Stimulus and TMT in red) on the left column, highlighting statistical difference. Each row displays the total power for a different frequency band, and in each graph, there corresponds the measures on the three channels considered: piriform cortex, hippocampal formation, and amygdaloid complex. The total power was calculated considering epochs of 5 min for the No Stimulus group, following the same protocol as the Stimulated groups, and considering the first afterdischarge at the 21st stimulus for the Stimulus group. The only statistical difference found was on the Theta band for the Stimulus group in the hippocampal formation. Mann–Whitney test, *p < 0.05.

**Figure 7:**
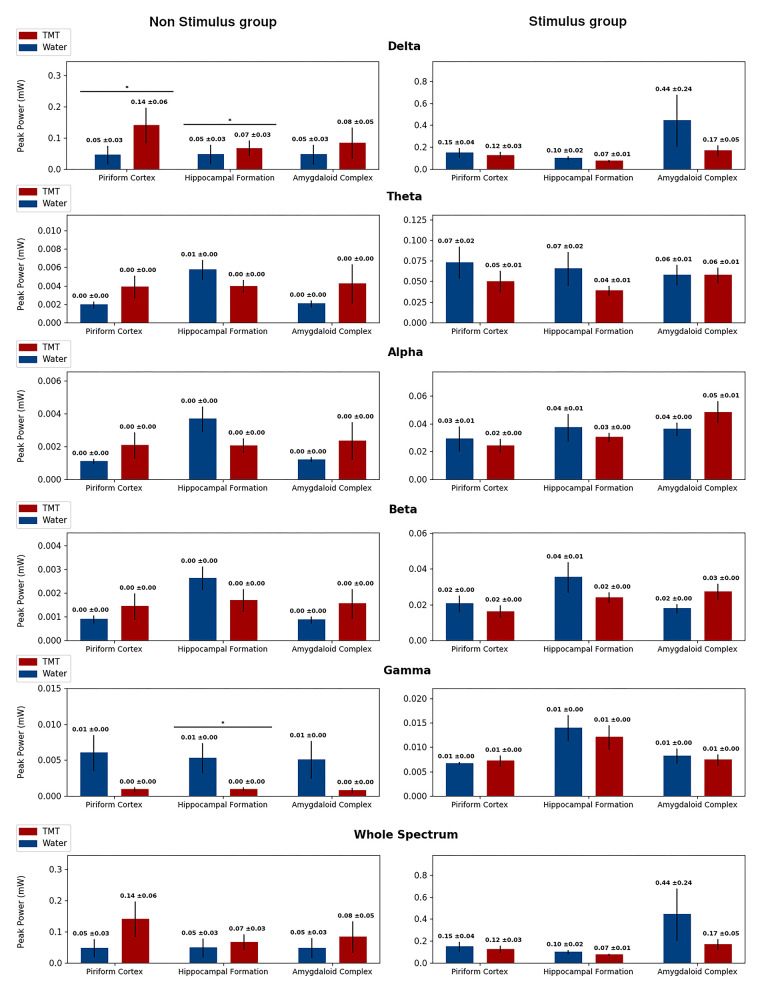
Peak power separated in band frequencies for the No Stimulus groups (No stimulus and Water in blue, and No stimulus and TMT in red) on the right column and from the Stimulus groups (Stimulus and Water in blue, and Stimulus and TMT in red) on the left column, highlighting statistical difference. Each row displays the Peak Power for a different frequency band, and in each graph, there corresponds the measures on the three channels considered: piriform cortex, hippocampal formation, and amygdaloid complex. The peak power was calculated considering epochs of 5 min for the No Stimulus group, following the same protocol as the Stimulated groups, and considering the first afterdischarge at the 21st stimulus for the Stimulus group. The only statistical difference found was for the No Stimulus group on the Delta band in the piriform cortex and hippocampal formation, and on the Gamma band in the hippocampal formation. Mann–Whitney test, *p < 0.05.

Spectral analysis indicated low-frequency activity on the No Stimulus and TMT group compared with the No Stimulus and Water group (Figures 6, 7 and Supplementary Figures 1, 2), with statistical difference for median frequency in the delta band on HIP (*p* > 0.05; Mann–Whitney test; Supplementary Figure 1) and AMYG (*p* > 0.05; Mann–Whitney test; Supplementary Figure 1), similar to findings for (1) frequency at the peak power in the beta band for AMYG (*p* > 0.05; Mann–Whitney test; Supplementary Figure 2) and (2) median frequency (Supplementary Figure 1) and peak power (Figure 7) both on the gamma band for HIP (*p* > 0.05; Mann–Whitney test). Finally, animals from the No Stimulus and TMT group presented lower median frequency in relation to its control (No Stimulus and Water group), considering the whole spectrum, on all channels (*p* > 0.05; Mann–Whitney test; Supplementary Figure 1). Thus, and as cited, these data curiously revealed that TMT significantly decreased oscillation in the different sub-bands, with the exception of peak power in delta (Figure 7).

More importantly, FFT EEG analysis (21st stimulus) showed that TMT decreased the total power in theta oscillations on HIP (*p* > 0.05; Mann–Whitney test), compared to the Stimulus and Water group (Figure 6). These findings corroborate the behavioral data that indicated that TMT interfered in the EEG synchronization typical of seizure activity.

## Discussion

As expected, the ARK model in Wistar rats induced a progressive increase in both behavioral and EEGraphic seizure activity, in accordance with previous data from our group (34). Furthermore, we interpreted in the detailed behavioral analysis (neuroethology) that TMT triggered fear/aversion-like reactions, which activated natural exploration for clues that indicated the location of the potential predator. In this context, and as a completely new finding, the olfactory exposure to TMT of ARK animals significantly reduced the seizure severity (Racine's scale). Both the seizure-TMT effect and the seizure-control effect were clearly evidenced by the behavioral sequential analysis.

EEGraphic evaluations indicated that TMT suppressed the activity of limbic epileptogenic network, given the reduction in occurrence of the first and second EEG afterdischarges. Moreover, FFT EEG analysis (21st stimulus) showed that TMT decreased the theta oscillations on HIP at Stimulus and TMT group, compared to its control. Surprisingly, our data also indicated low-frequency activity in the spectral features, particularly for HIP and AMYG, for No Stimulus and TMT group.

In fact, the ARK protocol as well as the classical amygdala kindling resulted in concomitant (in ictal and post-ictal periods) behavioral alterations (Figures 4A,B, 5A,B), associated to EEGraphic progression (afterdischarge, Figure 5), involving initially, as suggested by the literature, epileptogenic alterations in local circuits with synchronic and hyperactive neurons, which during the stimuli (acquisition of kindling) induce the recruitment of other brain areas (in our case, especially brainstem structures), resulting in progressive increase of severity (Figures 4A,B, 5A,B), in addition to increase in complexity of the afterdischarges (Figure 5) (5, 23, 25, 27, 28, 31, 34, 41, 45–48).

However, we verified in some seizures that the epileptiform activity did not necessarily accompany the behavioral changes, particularly when the second EEG afterdischarge was present only in the HIP. In fact, a possible explanation is that the Racine's scale and also the detailed behavioral sequential analysis measure the seizure activity, based on motor behavior (movement changes), while the EEG evidence small changes in seizure activity, not always associated (correlated) to behavioral expression (49, 50).

Another aspect observed in this study was the presence of Wet Dog Shakes (WDS) often correlated with the suppression of ARK-dependent seizures (particularly in Figure 4D, compared to Figure 4C, both POST-period). In fact, in a study from our laboratory comparing several experimental models of epilepsy, we described instances where WDS were predictive of seizure worsening and in others of seizures blockade (51). Also, the presence of the behavioral pattern (cluster) of grooming was associated with the reduction in seizure severity (Figure 4D, compared to Figure 4C, both POST-period). In agreement with this observation, in previous studies also from our group, we described a “hypergrooming” pattern, a model of compulsion induced by bilateral microinjection of oxytocin (OT) into the central nucleus of amygdala (52), and at the peak of this OT-induced hypergrooming, ARK can be blocked (personal communication; unpublished observations), probably another proof of “circuit antagonism” with effective anticonvulsant effects such as those discussed in the current study.

More importantly, we also found a significant reduction in the severity of the limbic seizures in the animals from the Stimulus and TMT group, in relation to the control group (Stimulus and Water group, Figures 3C,D). It is intriguing that we also found a reduction in LI (observed through Racine's scale), while not significant for animals exposed to Water (Stimulus and Water group, detailed in Materials and Methods), suggesting that Water or relative air humidity was not innocuous, possibly the air saturated with water vapor effect sensitized the respiratory airways, implicated as effects of acute stress (novelty or new environment), that physiologically could induce the grooming behavior (53), reinforcing the hypothesis previously cited.

Several evidences suggest a relationship between epilepsy and olfactory system (particularly “fear/aversion-related” network) based on a joint network activity, potentially related to “circuit antagonism” or “competition of systems” (17), via PC-AMYG-HIP. According to this proposal, the prior direct or indirect activation (by TMT) of brain areas (PC-AMYG-HIP) involved in seizure generation, recruitment or progression, could decrease the seizure activity or/and could temporary block the propagation of seizures, interfering in the communication between ictogenic structures, reducing a possible network-wise synchronization and the amplification of paroxysmal activity (54).

In this perspective, the processing of olfactory stimulus is distributed across a wide range of cerebral structures [for peripheral processing, in particular odorant receptors activated by TMT, see (55)], among them, the cortical amygdala, and then cortical and subcortical areas (56). Considering these intricate anatomical connections or relations of the temporal lobe and of the olfactory system (7, 8, 17, 57–59), more specifically, of the areas involved in the TMT processing (56, 60–63), which are also the same critically involved in limbic seizures expression in the amygdala kindling model (5, 23, 25, 27, 28, 31, 32, 34, 45, 47, 48, 64), the olfaction could interfere in limbic seizures (8) and vice versa (65, 66).

Interestingly, this association between olfaction and limbic seizures was clearly evidenced by the behavioral sequential analysis, demonstrating anticonvulsant properties, observed by the reduction of limbic behaviors at the STIMULATION period, with the presence of only one limbic behavior (EB) in the Stimulus and TMT group (Figure 4D), in contrast to five limbic behaviors (EB, MYO_1_, MYO_g_, MYO_h_, and REAR) in the Stimulus and Water group (Figure 4C), and at the POST-period, with the presence of only two limbic behaviors (EB and MT) for the Stimulus and TMT group (Figure 4D), in comparison to six limbic behaviors (EB, MT, MYO_1_, MYO_g_, MYO_h_, and REAR), added to GL, for the Stimulus and Water group, all observed on the 21st stimulus (Figure 4C). Additionally, TMT triggered the expression of classic fear/aversion-like behaviors (AR, FR, RU, and YA, Figure 1C), which potentially encouraged the natural exploration for clues that indicated the location of the predator (No stimulus and TMT group; Figure 1C), suggesting the activation of circuits involved in defensive responses, while animals of the Stimulus and TMT group present less robust fear/aversion-like behaviors, such as defensive IM, EXC, WI, and YA (Figure 4D). This situation may be a consequence of the antagonistic circuit activation that possibly inhibited the expression of typical fear/aversion behaviors, such as AR, FR, and RU, present in the No Stimulus and TMT group (Figure 1C). As a whole, these data suggest a competition of systems or circuit antagonism, with consequent suppression of seizure activity/propagation, as suggested by Efron (17), based on an endogenous inhibitory mechanism, or “natural” method of seizures control, that does not involve drug therapy or brain manipulations, functioning as a countermeasure in seizure activity (8, 15, 17). On the other hand, in other situations with seizures already initiated, the olfactory stimulation could cause widespread desynchronization (7, 8, 58).

As mentioned, it was described that the TMT, as well as TOL, induced rhythmical fast oscillations (8–21 Hz rhythmical potentials) in the PC, in contrast to ammonia (35). Recently, another study demonstrated olfactory bulb–hippocampus integration related to theta phase/gamma amplitude during exploration of home-cage odors, while TMT had caused avoidance in the spatial odor task (67). Our data showed a decrease of the theta band on HIP in the Stimulus and TMT group, compared to the Stimulus and Water group (Figure 6), which could correspond to a temporary block of the propagation or generalization of epileptic seizures (with the HIP exerting its gatekeeper function), since this band is associated to typical ictal pattern (68). These spectral data corroborate the reduction of the occurrence of first and second EEG afterdischarges (Figure 5). As a whole, our data suggest that TMT appears to have suppressive effects in the ARK-evoked epileptiform activity. Moreover, surprisingly, our data indicated low-frequency activity observed by FFT analysis, particularly in HIP and AMYG, for the No Stimulus and TMT group (Figures 6, 7 and Supplementary Figures 1, 2). Compared with faster frequencies, slow oscillatory rhythms are associated with the integration of distant neural assemblies and a more extensive propagation of far-field potential inside the brain's conducting media (electrical field potential). Therefore, the aversive sensation (TMT) in naive animals (no stimulus) could induce information processing in several distributed neural groups, demanding remote and precise time integration that reflects as slower oscillatory frequencies (toward the delta band). Meticulously triggered synchronization of several brain substrates, along specific time patterns, induced from the complex processing of the TMT stimuli, could interfere with endogenous control originating from an ictogenic attractor (in the current case, ARK-driven). In the same vein, a strong connection across different structures could also decrease the influence of one distinct pathological neural circuit in the surroundings; i.e., the spreading of ictal oscillation (seizure generalization) would be suppressed or avoided.

Another possible explanation for our results is based on the anticonvulsant effect of norepinephrine (69–77). In fact, TMT is a relevant ethological stressor to the rat, capable of activating physiological and behavioral responses triggered by the sympathetic-adreno-medullar (SAM) axis, which leads mainly to the release of catecholamines (78–80), among them norepinephrine. Horii et al. (81) demonstrated that urethane-anesthetized rats submitted to TMT had increased adrenal sympathetic nerve activity. This autonomic change is described in other studies (71, 76). In general, the norepinephrine depletion contributes to predisposition to seizures in the kindling model (70, 71, 76, 77), while normal/control doses may have anticonvulsant effect (69, 70, 72, 77). Feinstein et al. (73) suggested the effectiveness of stimulation of locus coeruleus in seizure control, in two patients with epilepsy (one generalized tonic–clonic seizure and one psychomotor seizure), demonstrated by reduction in frequency and severity of both types of seizures. On the other hand, it is also mentioned that TMT induces increase in corticosterone and corticotropin-releasing hormones (82–84). Additionally, other studies describe that TMT in high concentration can also activate the trigeminal nerve (85–88) and thus reduce seizure susceptibility, as anticonvulsant therapy. However, in accordance with our experimental design (as cited, the animals were exposed to water or TMT for 20 s, and immediately after, they were submitted to test stimulus; see details in Materials and Methods), we hypothesize the predominant effect of activation of the SAM system (mediates short-term effects), over the hypothalamus–pituitary–adrenal axis (HPA, short- and long-term effects) (79, 80), which can be added or not to possible trigeminal effect.

In summary, it is our view that TMT appears to act as an anticonvulsant substance through the combination of several factors that potentially include (1) antagonism or competition between “fear- and/or aversion-related networks” and “seizure-related networks” (current data), (2) noradrenergic effect, and (3) trigeminal nerve stimulation/activation (additional literature data). Such actions are highly complex (activation of the same anatomical structures with different functionality), non-linear, and immediate, so the deepening of the theme is fundamental for understanding the whole process.

Some issues deserve consideration; Lunardi et al. (89) suggest that the same olfactory stimulus can trigger or inhibit seizures in humans—depending on the state of activation of the neuronal membrane and also the state of cortical activation at the moment that the stimulus is given. Thus, further studies should evaluate the precipitation or inhibition switching of seizures by olfactory stimulation, in addition to the exposure effects, as duration and intensity. Furthermore, as future perspective, we wish to implement molecular solutions and cutting-edge technologies, for example optogenetics, which may confirm the mechanisms and the circuits responsible for the anticonvulsant effect of TMT.

Taken together, the results of this study are associated to the multinational research consortium *Epilepsies with External Modification of Ictogenesis* (*EpExMo*), aimed at collaborating with the knowledge associated to basic mechanisms and clinical features of epilepsy inhibited or triggered by external sensory or cognitive stimuli. Our current experiments are in fact absolutely coherent with previous studies (7, 8, 15–18, 20, 57, 90–92), adding consistent data to the literature. Furthermore, our findings certainly represent a partial component that will collaborate to the understanding of efficacy of odorants in the seizure response, with potential translational impact.

In conclusion, we showed that both behavioral and EEGraphic evaluations indicated that TMT, a potent molecule with strong biological relevance, in fact, “predator odor,” suppressed the epileptiform activity in limbic networks.

## Data Availability Statement

The raw data supporting the conclusions of this article will be made available by the authors, without undue reservation.

## Ethics Statement

The animal study was reviewed and approved by Commission for Ethics in Animal Experimentation (CETEA), at the University of São Paulo (protocol number: 200/2011).

## Author Contributions

PD-P and FD performed experiments. PD-P, JO, DM, DC, MM, JR, and EM analyzed the data. PD-P, PB-D, VS, and NG-C conceived and designed the study. PD-P, PB-D, DM, DC, VS, MM, and NG-C wrote, reviewed, and contributed to the final manuscript. All authors contributed to the article and approved the submitted version.

## Conflict of Interest

The authors declare that the research was conducted in the absence of any commercial or financial relationships that could be construed as a potential conflict of interest.

## Supplementary Material

Supplementary Figure 1 Median Frequency separated in band frequencies for the No Stimulus groups (No stimulus and Water in blue, and No stimulus and TMT in red) on the right column, and from the Stimulus groups (Stimulus and Water in blue, and Stimulus and TMT in red) on the left column, highlighting statistical difference. Each row displays the Median Frequency for a different frequency band, and in each graph there corresponds the measures on the three channels considered: Piriform Cortex, Hippocampal Formation and Amygdaloid Complex. The Median Frequency was calculated considering epochs of 5 min for the No Stimulus group, following the same protocol as the Stimulated groups, and considering the first afterdischarge at the 21st stimulus for the Stimulus group. Statistical differences were found only for the No Stimulus group on the Delta band in the Hippocampal Formation and the Amygdaloid Complex, on the Gamma band in the Hippocampal Formation, and on the Whole Spectrum on all channels. Mann Whitney test, ^*^*p* < 0.05.

Supplementary Figure 2 Frequency at Peak Power separated in band frequencies for the No Stimulus groups (No stimulus and Water in blue, and No stimulus and TMT in red) on the right column, and from the Stimulus groups (Stimulus and Water in blue, and Stimulus and TMT in red) on the left column, highlighting statistical difference. Each row displays the Frequency at Peak Power for a different frequency band, and in each graph there corresponds the measures on the three channels considered: Piriform Cortex, Hippocampal Formation and Amygdaloid Complex. The Frequency at Peak Power was calculated considering epochs of 5 min for the No Stimulus group, following the same protocol as the Stimulated groups, and considering the first afterdischarge at the 21st stimulus for the Stimulus group. Statistical differences were found only for the No Stimulus group on the Beta band in the Amygdaloid Complex. Mann Whitney test, ^*^*p* < 0.05.

## Abbreviations

AMYG: amygdaloid complex
ARK: amygdala rapid kindling
CETEA: Commission on Ethics on Animal Experimentation
EEGraphic: electroencephalographic
HIP: hippocampal formation
LI: limbic index
PC: piriform cortex
PRE: basal period or 5 min before of the electrical stimulus (except for the 21st stimulus, with a duration of 20 s)
POST: 5 min after electrical stimulation (or post-ictal period)
SEM: standard error of the mean
STIMULUS: period corresponding to electrical stimulation, with a duration of 10 s
TLE: temporal lobe epilepsy
TMT: 2,5-dihydro-2,4,5-trimethylthiazoline
TOL: toluene
WAR: Wistar Audiogenic Rat
χ^2^: chi-square: The complete list of acronyms of the behavioral items shown in the flowcharts and in the text is presented in Supplementary Table 1.
