# Supplementary figures and images for: Behavioral and EEGraphic Characterization of the Anticonvulsant Effects of the Predator Odor (TMT) in the Amygdala Rapid Kindling, a Model of Temporal Lobe Epilepsy
Source: Front Neurol. 2020 Nov 5;11:586724. doi: 10.3389/fneur.2020.586724 (PMC7674931; doi:10.3389/fneur.2020.586724)

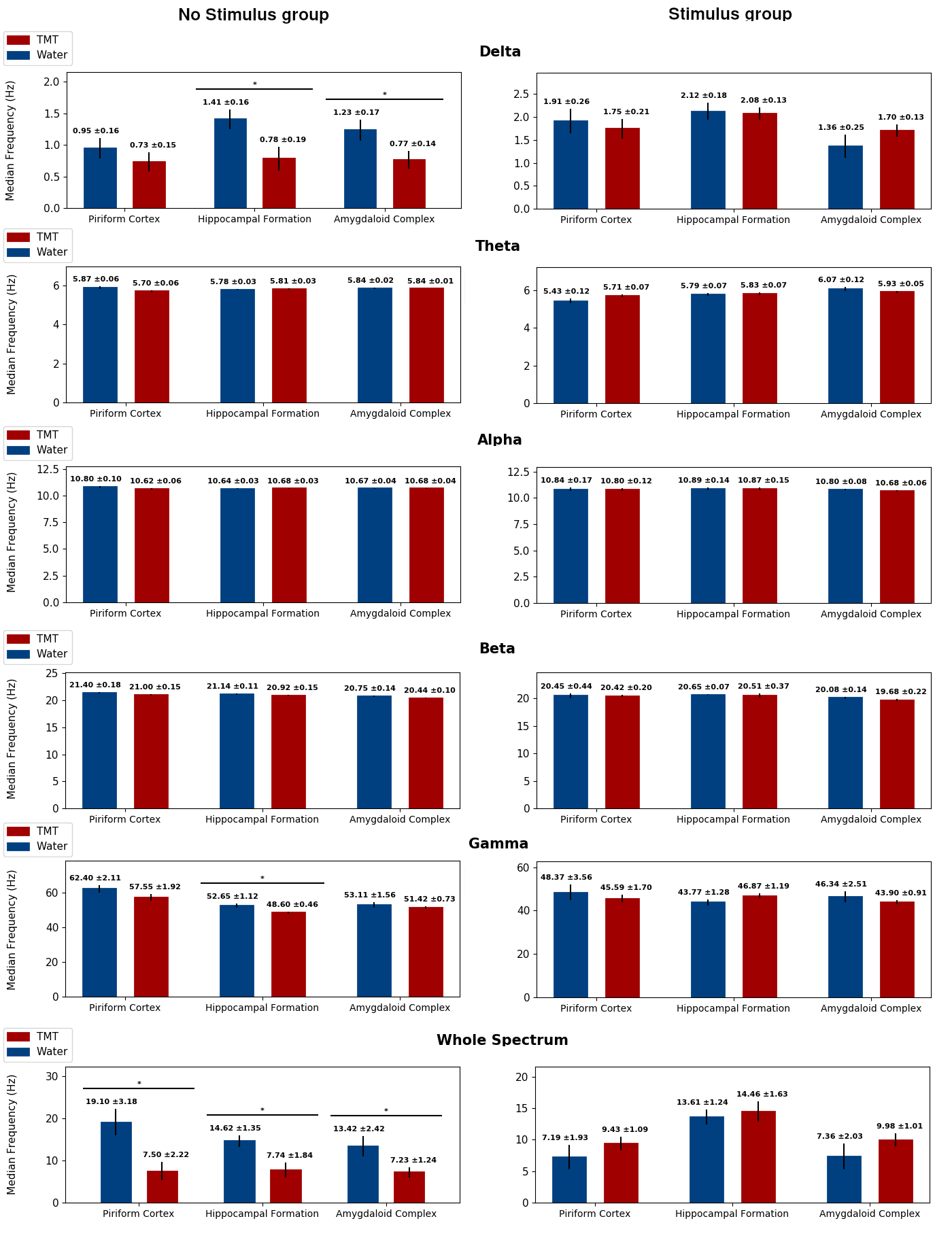

Supplement: Supplementary Figure 1 — Median Frequency separated in band frequencies for the No Stimulus groups (No stimulus and Water in blue, and No stimulus and TMT in red) on the right column, and from the Stimulus groups (Stimulus and Water in blue, and Stimulus and TMT in red) on the left column, highlighting statistical difference. Each row displays the Median Frequency for a different frequency band, and in each graph there corresponds the measures on the three channels considered: Piriform Cortex, Hippocampal Formation and Amygdaloid Complex. The Median Frequency was calculated considering epochs of 5 min for the No Stimulus group, following the same protocol as the Stimulated groups, and considering the first afterdischarge at the 21st stimulus for the Stimulus group. Statistical differences were found only for the No Stimulus group on the Delta band in the Hippocampal Formation and the Amygdaloid Complex, on the Gamma band in the Hippocampal Formation, and on the Whole Spectrum on all channels. Mann Whitney test, *p < 0.05. [file Image_1.TIFF]

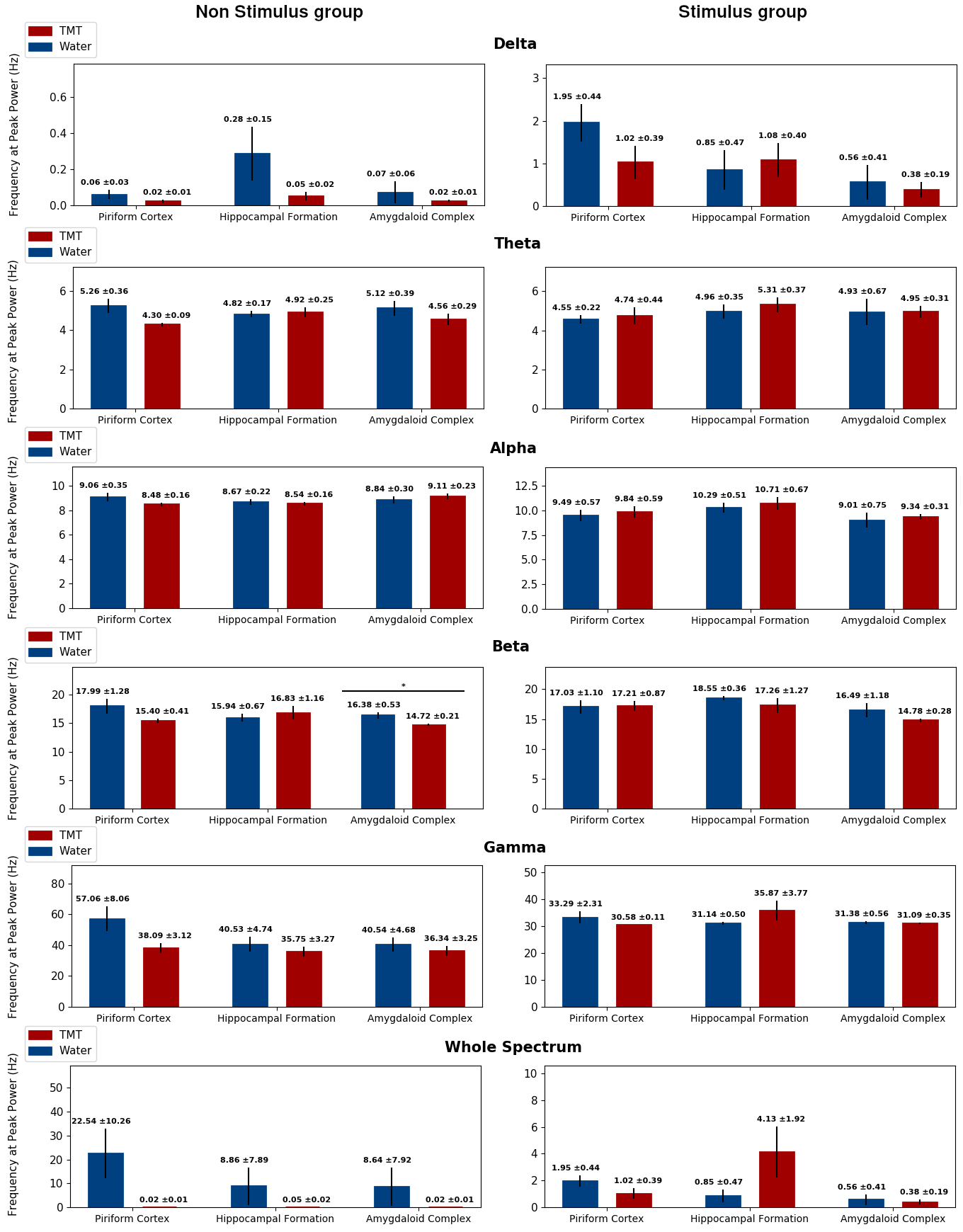

Supplement: Supplementary Figure 2 — Frequency at Peak Power separated in band frequencies for the No Stimulus groups (No stimulus and Water in blue, and No stimulus and TMT in red) on the right column, and from the Stimulus groups (Stimulus and Water in blue, and Stimulus and TMT in red) on the left column, highlighting statistical difference. Each row displays the Frequency at Peak Power for a different frequency band, and in each graph there corresponds the measures on the three channels considered: Piriform Cortex, Hippocampal Formation and Amygdaloid Complex. The Frequency at Peak Power was calculated considering epochs of 5 min for the No Stimulus group, following the same protocol as the Stimulated groups, and considering the first afterdischarge at the 21st stimulus for the Stimulus group. Statistical differences were found only for the No Stimulus group on the Beta band in the Amygdaloid Complex. Mann Whitney test, *p < 0.05. [file Image_2.TIFF]
