# Supplementary material for: Behavioral and EEGraphic Characterization of the Anticonvulsant Effects of the Predator Odor (TMT) in the Amygdala Rapid Kindling, a Model of Temporal Lobe Epilepsy
Source: Front Neurol. 2020 Nov 5;11:586724. doi: 10.3389/fneur.2020.586724 (PMC7674931; doi:10.3389/fneur.2020.586724)
Supplement: Supplementary file 3 [file Table_1.DOCX]

| **Supplementary Table 1.** Dictionary of some of the behavioral items and their corresponding acronyms, observed in this study (GARCIA-CAIRASCO et al., 1992) | |
| --- | --- |
| **Acronyms** | **English Description** |
| AF | Atonic Falling |
| AR | Arousal |
| AS | Abdominal Spasms |
| EB | Eye Blinking |
| ER | Erect Posture |
| EXC | Excretion of Feces |
| EXT | Extension |
| FALL | Falling |
| FR | Freezing |
| GN | Gnawing |
| GL | Gyrating, Left |
| GR | Gyrating, Right |
| GRF | Grooming of Face |
| GRG | Grooming of Genitals |
| GRH | Grooming of Head |
| GRR | Grooming of Body, Right |
| GRL | Grooming of Body, Left |
| IM | Immobility |
| JP | Jumping |
| LCL_1_ | Licking of Claws Left, forelimbs |
| LCL_2_ | Licking of Claws right, hindlimbs |
| LCR_1_ | Licking of Claws right, forelimbs |
| LCR_2_ | Licking of Claws right, hindlegs |
| LI | Licking |
| LIC | Licking of Claws |
| LY | Lying Posture |
| MT | Masticatory Movements |
| MYO_1_ | Myoclonus Spasms, forelimbs |
| MYO_2_ | Myoclonus Spasms, hindlimbs |
| MYOg | Generalized Myoclonus |
| MYO_h_ | Myoclonus Spasms, Head |
| NOD | Nodding |
| PIM | Postictal Immobility |
| PIV | Pivoting |
| REAR | Rearing |
| RU | Running |
| SAL | Salivation |
| SC | Scanning |
| SCRL | Scratching of Body, Left |
| SCRR | Scratching of Body, Right |
| SH | Head Shaking |
| SN | Sniffing |
| STA | Startle |
| TNBL | Tonic Neck and Body Turning Left |
| WA | Walking |
| WDS | Wet Dog Shaking |
| WI | Withdraw |
| YA | Yawning |
