# Supplementary material for: Behavioral and EEGraphic Characterization of the Anticonvulsant Effects of the Predator Odor (TMT) in the Amygdala Rapid Kindling, a Model of Temporal Lobe Epilepsy
Source: Front Neurol. 2020 Nov 5;11:586724. doi: 10.3389/fneur.2020.586724 (PMC7674931; doi:10.3389/fneur.2020.586724)
Supplement: Supplementary file 4 [file Table_2.DOCX]

| **Supplementary Table 2.** *p*-value during the POST stimulus period, indicating the evolution of Rapid Amygdala Kindling of Figure 3B | | | |
| --- | --- | --- | --- |
| 1^st^ *versus* 16^th^ | * | 3^rd^ *versus* 19^th^ | * |
| 1^st^ *versus* 19^th^ | ** | 4^th^ *versus* 19^th^ | * |
| 2^nd^ *versus* 12^th^ | * | 5^th^ *versus* 12^th^ | * |
| 2^nd^ *versus* 14^th^ | * | 5^th^ *versus* 14^th^ | * |
| 2^nd^ *versus* 15^th^ | * | 5^th^ *versus* 15^th^ | * |
| 2^nd^ *versus* 16^th^ | ** | 5^th^ *versus* 16^th^ | ** |
| 2^nd^ *versus* 18^th^ | * | 5^th^ *versus* 18^th^ | * |
| 2^nd^ *versus* 19^th^ | *** | 5^th^ *versus* 19^th^ | *** |
| 2^nd^ *versus* 20^th^ | * | 5^th^ *versus* 20^th^ | * |
| 3^rd^ *versus* 16^th^ | * | 9^th^ *versus* 19^th^ | * |
| Anova One Way, Fridman Test, Dunn’s Post Test: **p*<0.05; ***p*<0.01 and ****p*<0.001. | | | |
